# Sirt3 mitigates LPS‐induced mitochondrial damage in renal tubular epithelial cells by deacetylating YME1L1

**DOI:** 10.1111/cpr.13362

**Published:** 2022-11-26

**Authors:** Yonghong Jian, Yifei Yang, Lingli Cheng, Xueyan Yang, Hongyan Liu, Wei Li, Yuhan Wan, Dingping Yang

**Affiliations:** ^1^ Department of Nephrology Renmin Hospital of Wuhan University Wuhan China; ^2^ Nephrology and Urology Research Institute of Wuhan University Wuhan Hubei China

## Abstract

Acute kidney injury (AKI) is often secondary to sepsis. Increasing evidence suggests that mitochondrial dysfunction contributes to the pathological process of AKI. In this study, we aimed to examine the regulatory roles of Sirt3 in Lipopolysaccharide (LPS)‐induced mitochondrial damage in renal tubular epithelial cells (TECs). Sirt3 knockout mice were intraperitoneally injected with LPS, and cultured TECs were stimulated with LPS to evaluate the effects of Sirt3 on mitochondrial structure and function in TECs. Electron microscopy was used to assess mitochondrial morphology. Immunofluorescence staining was performed to detect protein expression and examine mitochondrial morphology. Western blotting was used to quantify protein expression. We observed that LPS increased apoptosis, induced disturbances in mitochondrial function and dynamics, and downregulated Sirt3 expression in a sepsis‐induced AKI mouse model and human proximal tubular (HK‐2) cells in vitro. Sirt3 deficiency further exacerbated LPS‐induced renal pathological damage, apoptosis and disturbances in mitochondrial function and dynamics. On the contrary, Sirt3 overexpression in HK‐2 cells alleviated these lesions. Functional studies revealed that Sirt3 overexpression alleviated LPS‐induced mitochondrial damage and apoptosis in TECs by promoting OPA1‐mediated mitochondrial fusion through the deacetylation of i‐AAA protease (YME1L1), an upstream regulatory molecule of OPA1. Our study has identified Sirt3 as a vital factor that protects against LPS‐induced mitochondrial damage and apoptosis in TECs via the YME1L1‐OPA1 signaling pathway.

## Graphical abstract

In a physiological state, Sirt3 has a certain deacetylation effect on YME1L1, and YME1L1 deacetylation can promote OPA1‐mediated mitochondrial fusion, so that mitochondrial fusion and fission are in a balanced state. When sepsis‐induced AKI occurs, the expression of Sirt3 is reduced, resulting in a decrease in the deacetylation level of YME1L1 and a decrease in the expression of L‐OPA, which in turn reduces mitochondrial fusion and increases fission, ultimately leading to mitochondrial dysfunction and apoptosis.

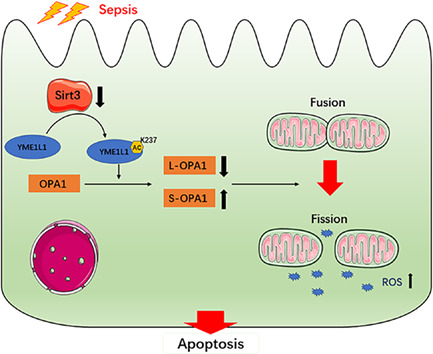

## 1 INTRODUCTION

Sepsis is defined as organ dysfunction caused by a dysregulated deleterious response to infection.^1^ Studies have shown that sepsis is the leading cause of death in ICU patients without heart disease.^2^ The kidney is among the first organs to be affected by sepsis. Acute kidney injury (AKI) occurs in approximately 50% of patients with sepsis, and the mortality rate can be as high as 60%.^3^, ^4^ Lipopolysaccharide (LPS) is a unique chemical component in the outer wall of gram‐negative bacteria and is one of the major causes of sepsis. Sepsis‐induced AKI animal models established by intraperitoneal injection of LPS have been widely used in basic research.^5^ The pathological phenotype of AKI is characterized by proximal tubular injury.^6^ Although the pathogenesis of septic AKI has not been fully elucidated, disturbances in energy metabolism in tubular epithelial cells (TECs) have been recognized as one of the important mechanisms of septic AKI in recent years.^7^

As the energy metabolism centre of cells, mitochondria provide energy by generating ATP through oxidative phosphorylation (OXPHOS).^8^ The precise and complex network of antioxidants and mitochondrial quality control are essential for cellular homeostasis.^9^ Excessive extracellular stress contributes to mitochondrial homeostatic imbalance, which leads to disturbances in energy metabolism and ultimately cell death.^10^ Fusion and fission homeostasis are the most important links in mitochondrial quality control, which is also known as mitochondrial dynamics.^11^ Mitochondrial dynamics are the dynamic equilibrium of fission and fusion and are regulated by a series of proteins.^12^ Dynamin‐related protein (Drp1) and fission protein 1 (Fis1) mainly mediate mitochondrial fission, and mitochondrial fusion protein 1 and 2 (MFN‐1, MFN‐2) and optic atrophy protein 1 (OPA1) mainly mediate mitochondrial fusion.^13^ OPA1 is hydrolysed and regulated by two mitochondrial proteases, OMA1 and YME1L1 and exists as long (L‐OPA1) and short (S‐OPA1) forms. L‐OPA1 is mainly responsible for inner mitochondrial membrane fusion and maintains normal mitochondrial function. In contrast, the accumulation of S‐OPA1 contributes to mitochondrial fission.^14^ Studies have shown that YME1L1 is a key enzyme in stabilizing L‐OPA1.^15^ The kidney is a mitochondrion‐rich energy metabolic organ that possesses widely distributed mitochondria in the proximal tubules. Accumulating evidence suggests that abnormal mitochondrial dynamics are associated with a variety of pathologic processes, including AKI.^16^ Silent mating type information regulator 2 (SIR2), or sirtuins, are NAD+‐dependent class III histone deacetylases that maintain mitochondrial homeostasis and antioxidant ability.^17^ Sirtuin 3 (Sirt3) has been shown to have powerful deacetylation effects on many metabolic targets^18^ and distinct protective effects on various diseases.^19^, ^20^ Studies have revealed that Sirt3 protein levels are downregulated in AKI animal models induced by LPS or cisplatin, and increased Sirt3 expression alleviates tubule injury.^21^, ^22^, ^23^ Although Zhao et al.^24^ demonstrated that Sirt3 protected the kidney by reducing oxidative stress and inflammation in a sepsis‐induced AKI mouse model based on caecal ligature and puncture, the molecular mechanisms of Sirt3 in sepsis‐induced AKI have not been fully illustrated and need to be clarified.

In this study, a mouse model of septic AKI induced by LPS and human proximal renal TECs cultured in vitro in the presence of LPS were used to observe the effects of LPS stimulation on tubular damage, apoptosis, mitochondrial function and morphology and Sirt3 expression. Abnormalities in mitochondrial function and dynamics during septic AKI were confirmed. Furthermore, Sirt3‐knockout mice and Sirt3‐overexpressing HK‐2 cells were used to construct a septic model and further observe the changes in mitochondrial function and dynamics, as well as the activity levels of upstream related molecules, to evaluate the role of Sirt3 in mitochondrial injury in TECs in septic AKI and the mechanism. The present study aimed to examine the effects of Sirt3 on mice with septic AKI to provide new insights into the pathogenesis and clinical treatments.

## 2 MATERIALS AND METHODS

### 2.1 Reagents and antibodies

LPS was purchased from Sigma (MO, USA). Anti‐Sirt3, anti‐Cyt‐c and anti‐acetylated lysine were obtained from Cell Signalling Technology (MA, USA). Immunofluorescent anti‐Sirt3 was purchased from Santa Cruz (CA, USA). Anti‐OPA1, anti‐Drp1, anti‐VDAC, anti‐TOM‐20 and anti‐GAPDH were purchased from Abcam (MA, USA). Anti‐Bax and anti‐YME1L1 were purchased from Proteintech (Wuhan, China). Anti‐Flag, Alexa Fluor 488‐ and 594‐conjugated fluorescent secondary antibodies and HRP‐conjugated secondary antibodies were purchased from Antgene (Wuhan, China).

### 2.2 Animals

SIRT3‐knockout C57BL/6 heterozygous mice were purchased from Cyagen Biosciences Inc. (Certificate No. 44410400001307). Wild‐type (WT) C57BL/6 mice were crossed with Sirt3‐knockout heterozygous C57BL/6 mice to generate Sirt3‐knockout homozygous (Sirt3‐KO) mice. Conventional PCR was performed to identify genotypes. The mice were housed under SPF conditions with free access to water and food in the Centre of Animal Experiments at Renmin Hospital of Wuhan University. The septic AKI model was established in 8–10‐week‐old male mice weighing 20–26 g. LPS (10 mg/kg) was injected intraperitoneally, and saline served as the vehicle control. The mice were sacrificed after 24 or 48 h, and renal tissues and blood were harvested. All experiments were performed in compliance with related animal ethical regulations and approved by the Animal Ethics Committee of Renmin Hospital of Wuhan University (IACUC Issue No. 20211103).

### 2.3 Detection of serum creatinine and blood urea nitrogen

Harvested blood samples were allowed to settle for 2 h at room temperature and subsequently centrifuged to collect serum. Serum creatinine (Scr) and blood urea nitrogen (BUN) were measured with an automatic biochemical analyser at Renmin Hospital of Wuhan University.

### 2.4 Pathological and immunohistochemical staining

The kidney tissues were fixed overnight in 4% paraformaldehyde, embedded in paraffin and sectioned. The sections were dewaxed with xylene, hydrated in graded concentrations of ethanol and stained with haematoxylin–eosin (HE). For immunohistochemical (IHC) staining, the sections were dewaxed and hydrated in sequence, and then antigen retrieval was performed using sodium citrate buffer. Subsequently, the tissue sections were incubated with 5% normal goat serum for 20 min and incubated overnight at 4°C with anti‐Sirt3 primary antibodies (1:100). Then, a corresponding HRP‐labelled secondary antibody with DAB was used for the chromogenic reaction. Finally, images were captured under a light microscope (Olympus, Japan). The degree of tissue injury was scored as the percentage of damaged tubules (0–4 points).

### 2.5 Immunofluorescence staining

For immunofluorescence (IF) staining, the initial steps were the same as those for IHC staining until antigen repair. Accordingly, cell climbing slices were fixed for 20 min in 4% paraformaldehyde. Subsequently, corresponding primary antibodies (Sirt3 1:100, TOM‐20 1:100, YME1L1 1:100) were added and incubated after the samples were blocked with 5% BSA. Then, the sections or climbing pieces were incubated with fluorescent secondary antibodies. After the sections were stained with DAPI and sealed, images were captured with a confocal microscope (Olympus, Japan).

### 2.6 Genomic PCR

The toes were clipped and placed in lysis solution in a 55°C water bath for 4–5 h until the toes were dissolved. DNA was extracted using a DNA extraction kit. Next, the DNA sample was genotyped according to the PCR protocol using the following primers: forward (F1): 5′‐CAGTCAGTGACATCTTGGCTCTAC‐3′ and reverse (R1): 5‐CAGCCCAGCCTTATGTTCCTTTAC ‐3′. The PCR system was as follows: PCR mix, 12.5 μl; ddH2O, 9 μl; forward primer (F1), 1 μl; reverse primer (R1), 1 μl; DNA, 1.5 μl; and total volume, 25 μl. The PCR conditions were as follows: predenaturation at 94°C for 3 min, denaturation at 94°C for 30 s, annealing at 60°C for 35 s, extension at 72°C for 35 s and extension at 70°C for 5 min, and steps 2–4 were repeated for 38 cycles. Finally, the PCR products were subjected to agarose gel electrophoresis.

### 2.7 TUNEL assay

To analyse apoptotic TECs in proximal tubules, a TUNEL assay was performed according to the manufacturer's protocol (Roche Applied Science, Germany). Briefly, the sections were deparaffinized, rehydrated and then incubated with Proteinase K working solution for 15 min at 37 °C. Next, the TUNEL reaction mixture was added. After the sections were sealed, images were captured with a fluorescence microscope (Olympus, Japan).

### 2.8 Detection of reactive oxygen species

Reactive oxygen species (ROS) levels in renal tissues were detected by the fluorescent probe DHE (YEASEN, China). Harvested kidneys were used to prepare the frozen sections. The sections were incubated with DHE working solution at 37°C for 60 min and protected from light. Cellular ROS production was evaluated with the probe DCFH‐DA (Beyotime, China). After the sections were stained with DAPI and sealed, images were captured with a confocal microscope (Olympus, Japan).

### 2.9 Transmission electron microscopic analysis

Renal cortices were minced into 1 mm^3^ pieces and fixed with glutaraldehyde followed by osmic acid. Ultrathin sections were stained with lead citrate and alcoholic uranyl acetate for mitochondrial visualization and electron microscopy (TEM) imaging (Hitachi, Japan). The aspect ratio of the mitochondria was assessed using ImageJ software.

### 2.10 Cell culture and treatments

Human proximal TECs (HK‐2 cells) were stored in the laboratory. Cells were cultured in DMEM‐F12 medium (HyClone) with 10% FBS (HyClone) and 1% penicillin/streptomycin (HyClone) at 37°C in the presence of 5% CO2. The experimental group was stimulated with LPS (LPS4391, 10 μg/ml) for 24 h, and the control group was treated with the same amount of normal saline. Human embryonic kidney 293 (HEK‐293) cells were stored in the laboratory and cultured in high‐glucose DMEM (HyClone).

### 2.11 Cell transfection

For Sirt3 overexpression, when the cells (HK‐2) reached 50%–70% confluence, Lipofectamine 2000 reagent (Invitrogen) was used to transfect pcDNA3.1‐Sirt3 or pcDNA3.1 according to the manufacturer's protocol.

Human WT YME1L1, mutant YME1L1 K237R (deacetylation mimetic) and mutant YME1L1 K237Q (acetylation mimetic) plasmids were constructed as previously described^25^ and obtained from Ji Kai Company (Shanghai, China). Transient transfection was performed using the Lipofectamine 2000 reagent (Invitrogen).

### 2.12 Mitochondrial protein extraction

After the cells in each group were stimulated, the cells were scraped with a cell scraper, and the cell suspension was collected in an EP tube. According to the instructions, mitochondrial protein was extracted using a mitochondrial isolation kit (Thermo Scientific).

### 2.13 Western blotting

Renal tissues and cells were lysed in protein extraction buffer containing a protease inhibitor cocktail and PMSF and centrifuged at 13,000 rpm at 4°C for 5 min. The protein concentrations of the supernatants were quantified with a BCA protein assay kit (Pierce), mixed with loading buffer and boiled at 100°C for 5 min. The samples were separated by SDS–PAGE and then transferred to PVDF membranes. The membranes were blocked in 5% skim milk at room temperature for 1 h and incubated with the indicated primary antibodies (Sirt3 1:1000, Drp1 1:1000, OPA1 1:1000, YME1L1 1:1000, Bax 1:1000, Cyt‐c 1:1000, acetylated‐lysine 1:1000, Flag 1:1000, VDAC 1:1000 and GAPDH 1:1000) overnight at 4°C. HRP‐conjugated secondary antibodies were added to the membranes and incubated for 1 h before chemiluminescence analysis with ECL and ChemiDoc XR Plus (Bio‐Rad) was performed.

### 2.14 Mitochondrial membrane potential measurement

Changes in the mitochondrial membrane potential of HK‐2 cells treated with or without LPS were measured using a JC‐1 kit according to the manufacturer's instructions (Beyotime, China). Briefly, JC‐1 dye was added to the treated cells for 20 min at 37°C. The cells were washed twice with assay buffer and placed in culture medium before being captured with a fluorescence microscope.

### 2.15 Cell apoptosis assay

The slides of treated cells were fixed with 4% paraformaldehyde for 30 min, and then Hoechst 33342 staining solution (Beyotime, China) was added away from the light for 5 min. After being washed and sealed, the cells were captured with a fluorescence microscope.

To assess cell apoptosis by flow cytometry, treated cells were collected, resuspended in binding buffer and then stained with PE Annexin V and 7‐AAD (BD Biosciences). The cells were analysed by an Accuri flow cytometer (BD Biosciences). Flow‐Jo software was used to analyse the results.

### 2.16 Immunoprecipitation assay

Immunoprecipitation (IP) assays were performed according to the manufacturer's protocol (Beyotime, China). After the appropriate treatment, proteins were extracted from HK‐2 cells or mouse renal cortex and incubated with the indicated primary antibodies (acetylated‐lysine or Sirt3) and protein A+G agarose suspension beads (Beyotime, China) with rotating overnight at 4°C. The beads were centrifuged and washed three times, and then the immune complexes were eluted by boiling in a loading buffer. To determine the degree of YME1L1 acetylation and the interaction between Sirt3 and YME1L1, the proteins were separated by SDS–PAGE, and the blots were incubated with the indicated primary antibodies (YME1L1 1:1000) overnight at 4°C. Additional steps were the same as the protocols for western blotting.

### 2.17 Data analysis

All values are expressed as the means ± standard deviation and were analysed by SPSS 19.0 statistical software. Statistical comparisons were performed using a *t* test, and differences among multiple groups were compared using one‐way analysis of variance (ANOVA). *p* < 0.05 was considered significant.

## 3 RESULTS

### 3.1 Clinical and histopathological changes and Sirt3 expression in LPS‐induced septic AKI mice

**FIGURE 1:**
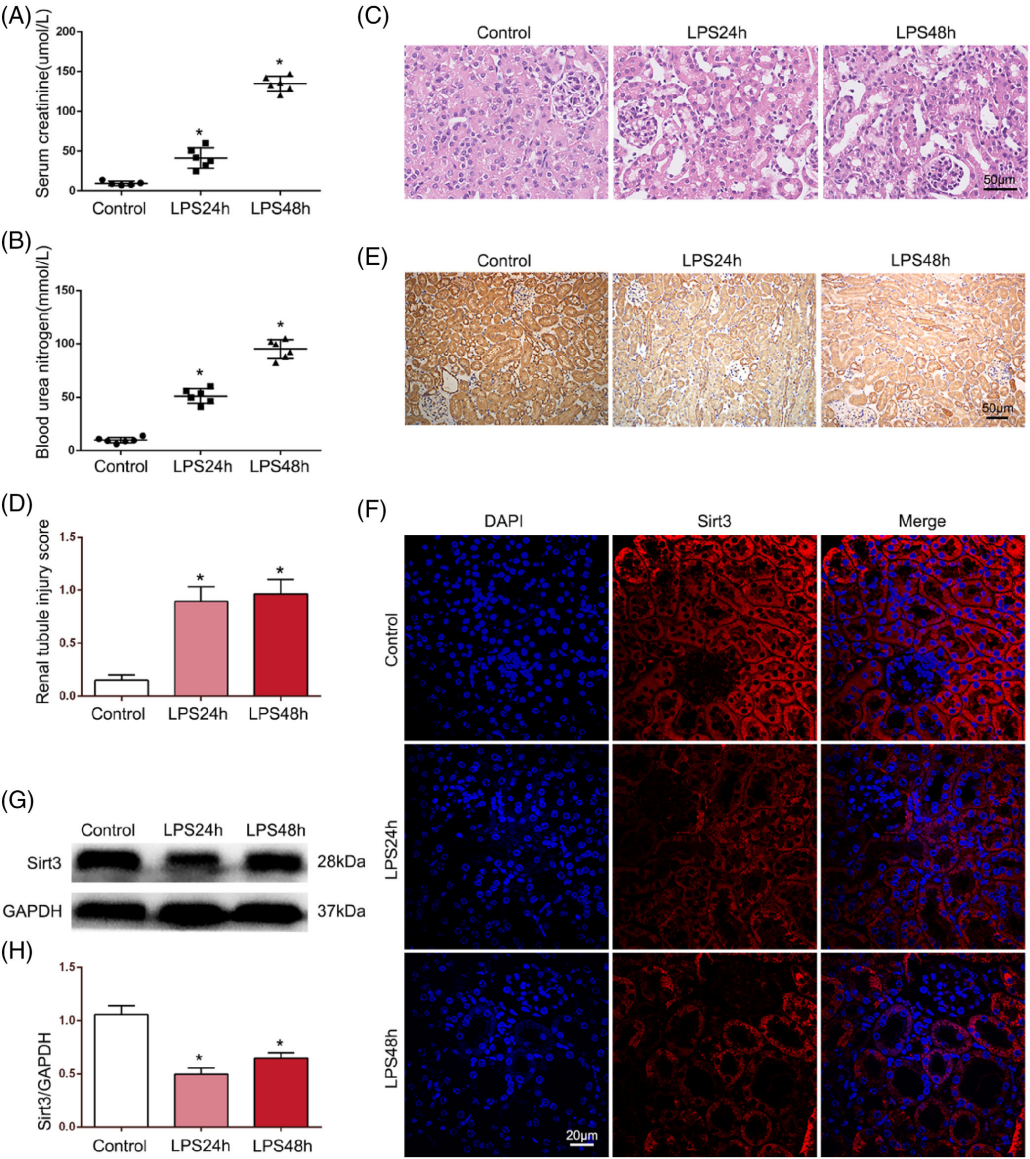
Effects of LPS on mouse kidney and Sirt3. (A, B) The serum biochemical indices of mice, including serum creatinine and blood urea nitrogen, were measured (*n* = 6). (C, D) HE staining was utilized to observe the renal morphology and the tubular damage score was performed (*n* = 6) (original magnification, ×400). Scale bar = 50 μm. (E) Immunohistochemical staining was performed to detect the expression of Sirt3 on kidneys (original magnification, ×200). Scale bar = 50 μm. (F) Immunofluorescence staining showed the expression and distribution of Sirt3 on kidney (original magnification, ×600). Scale bar = 50 μm. (G, H) Representative western blot of Sirt3 expression and western blot quantification in renal tissues (*n* = 6). *p < 0.05 compared with the control group.

Compared to those in the saline control group, the serum indices, including BUN and Scr, of LPS‐induced septic AKI mice were increased significantly at 24 h and were further increased at 48 h (Figure 1A,B). HE staining showed conspicuous pathological alterations over time, including severe vacuolar degeneration, detachment of TECs and tubular lumen dilatation (Figure 1C,D). IHC staining revealed that the intensity of Sirt3‐positive staining was weaker at 24 h, and after 48 h, the expression of Sirt3 was partially restored (Figure 1E). IF staining and western blotting showed results similar to those of IHC staining (Figure 1F,H). These results indicated that LPS acutely damaged the function and structure of the kidney in septic AKI and that Sirt3 was involved in these processes.

### 3.2 Effects of LPS on mitochondrial dynamics and function in mice

**FIGURE 2:**
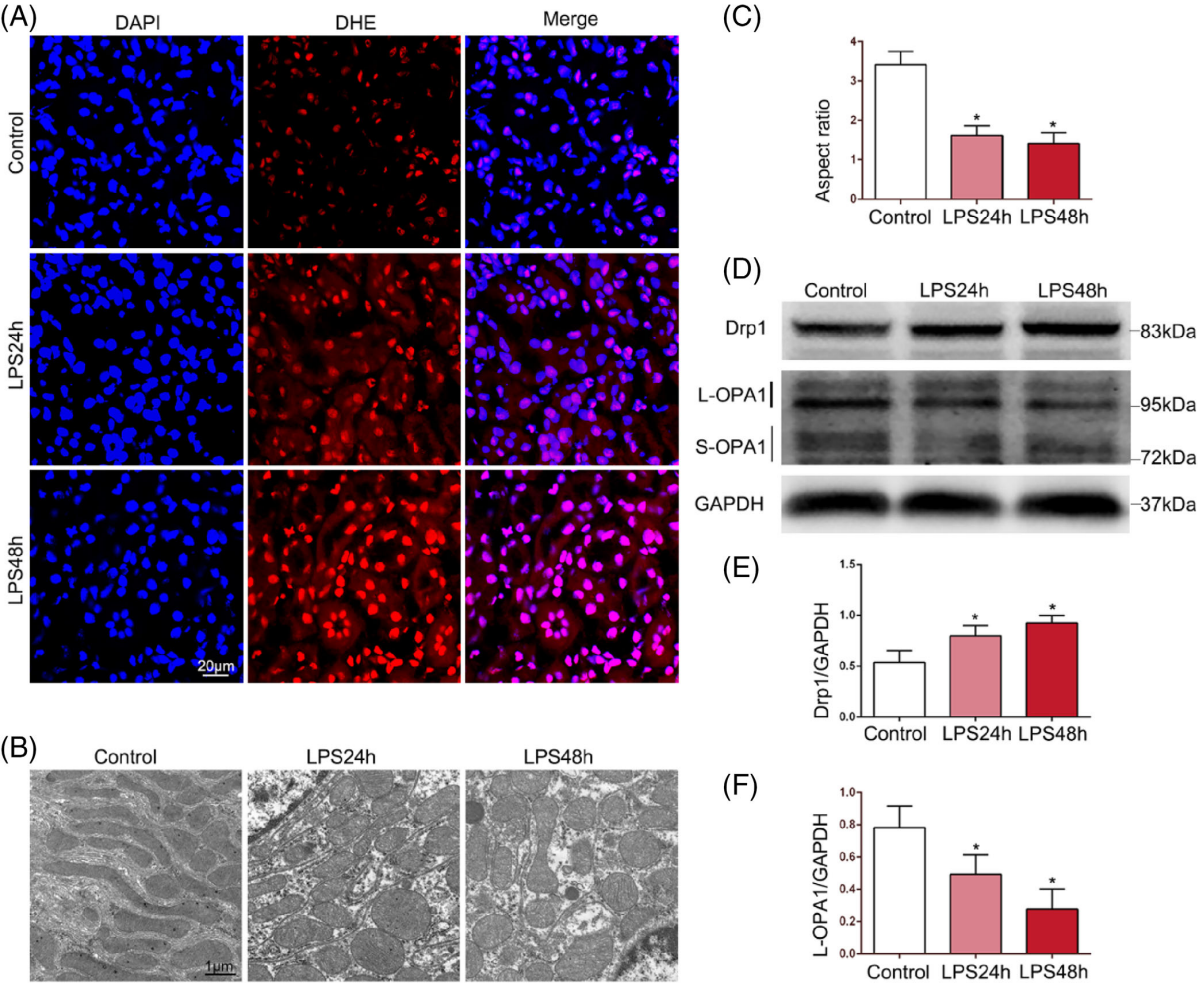
Effects of LPS on mitochondrial dynamics and functions of mice. (A) DHE staining evaluated ROS production in TECs of mice with time points. DAPI (blue) was used to stain the cell nucleus (*n* = 6) (original magnification, ×600). Scale bar = 20 μm. (B, C) The morphology of mitochondria of TECs in each group was observed by electron microscopy, and the aspect ratio of mitochondria was performed for semiquantitative evaluation (*n* = 6) (original magnification, ×8000). Scale bar = 1 μm. (D–F) Representative western blots of Drp1 and OPA1 expression and western blot quantification in renal tissues (*n* = 6). *p < 0.05 compared with the control group.

Oxidative stress levels and mitochondrial function were evaluated using DHE, an ROS‐specific fluorescent probe. The results revealed that after LPS stimulation, the level of ROS in TECs was significantly increased (Figure 2A). Electron microscopy demonstrated that TEC mitochondria in the control group were mostly long tubules, while those in the LPS group were round and short, and the mitochondrial aspect ratio was decreased, suggesting decreased mitochondrial fusion and increased fission after LPS stimulation (Figure 2B,C). The protein expression of Drp1 was upregulated with time, and L‐OPA1 levels were downregulated with time (Figure 2D,F). These results suggest that LPS leads to abnormal mitochondrial function and dynamics.

### 3.3 Effects of LPS on apoptosis, mitochondrial dynamics and Sirt3 expression in HK‐2 cells

**FIGURE 3:**
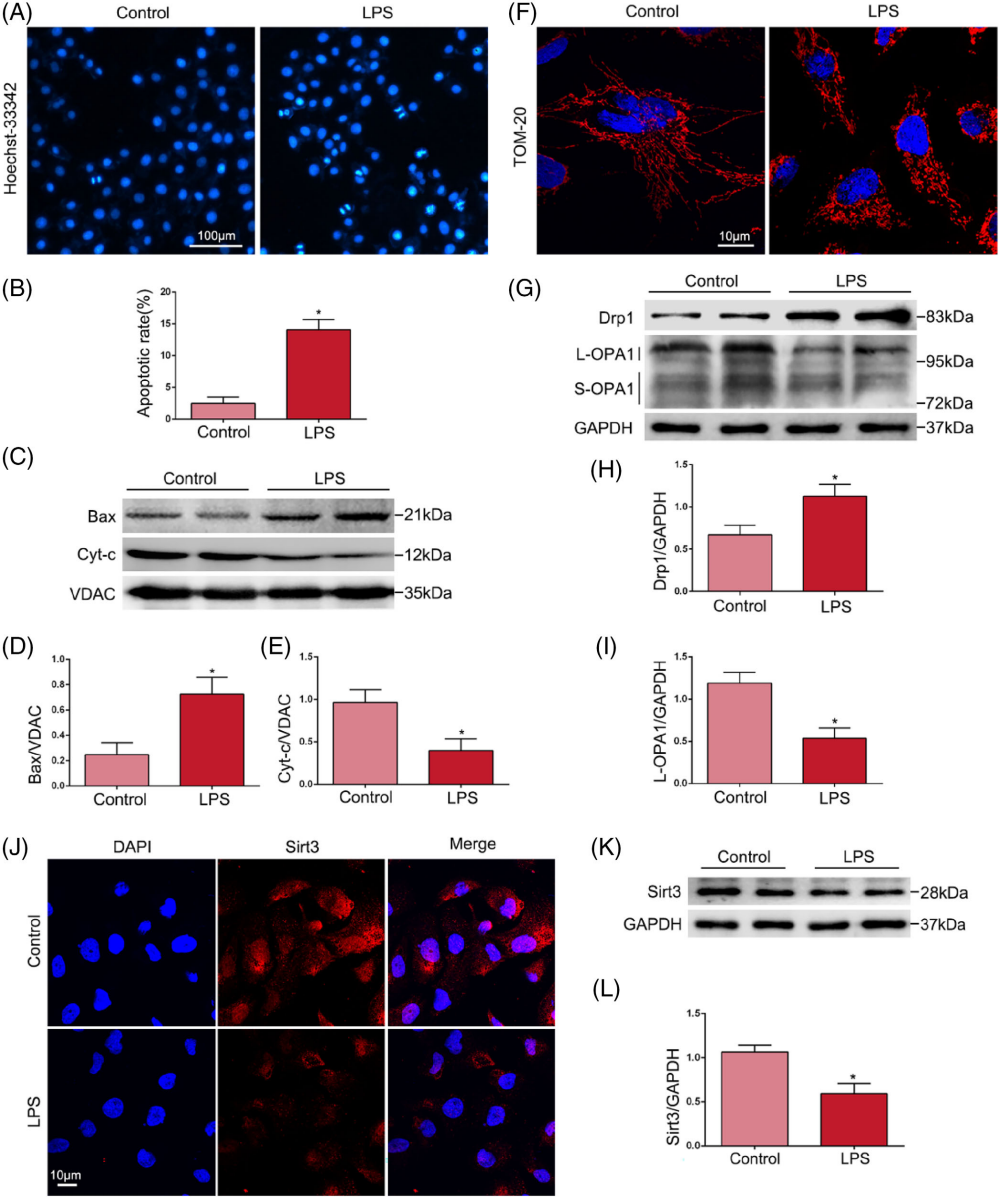
Effects of LPS on HK‐2 cells in vitro and Sirt3. (A, B) Cell apoptosis was evaluated by Hoechst‐33342 staining. Apoptotic cells were identified as those with fragmented and/or condensed nuclei (original magnification, ×200). Scale bar = 100 μm. (C–E) Representative western blots of Bax and Cyc‐t expression and western blot quantification in HK‐2 cells (*n* = 4). (F) Mitochondrial morphology in HK‐2 cells was subjected to IF‐staining using TOM‐20 antibody (original magnification, ×1000). Scale bar = 10 μm. (G–I) Representative western blots of Drp1 and OPA1 expression and western blot quantification in HK‐2 cells (*n* = 4). (J) The expression of Sirt3 was detected by IF staining (original magnification, ×600). Scale bar = 10 μm. (K, L) Representative western blot of Sirt3 expression and western blot quantification in HK‐2 cells (*n* = 4). *p < 0.05 compared with the control group.

To further examine the effects of LPS on TECs, cell models were developed using LPS‐induced HK‐2 cells. Apoptotic cells were identified as those with fragmented and/or condensed nuclei under Hoechst‐33342 staining, which was more obvious after LPS treatment (Figure 3A,B). Alternately, mitochondria were isolated from HK‐2 cells by a mitochondria isolation kit (Thermo Scientific), and the expression of the apoptosis‐related proteins Bax and Cyt‐c were examined by western blotting. The results revealed that LPS significantly induced the protein levels of Bax; in contrast, the expression of Cyt‐c was obviously downregulated (Figure 3C–E). This finding suggests that the mitochondrial apoptosis pathway of HK‐2 cells is activated by LPS stimulation. HK‐2 cells were subjected to IF staining using TOM‐20 antibodies (a mitochondrial marker). Normal cells exhibited an intact network of successive tubular mitochondria, while the mitochondria of cells that were stimulated with LPS formed fragmented and discontinuous networks (Figure 3F). Moreover, the protein expression of Drp1 in treated HK‐2 cells was significantly upregulated, and the expression of L‐OPA1 was decreased (Figure 3G–I). IF staining and western blotting showed that the expression of Sirt3 was decreased by LPS treatment (Figure 3J–L). These results suggested that LPS promoted apoptosis and abnormal mitochondrial dynamics in TECs and that Sirt3 played a major role in these processes.

### 3.4 Sirt3 deficiency exacerbates abnormal mitochondrial dynamics and function in septic AKI mice

**FIGURE 4:**
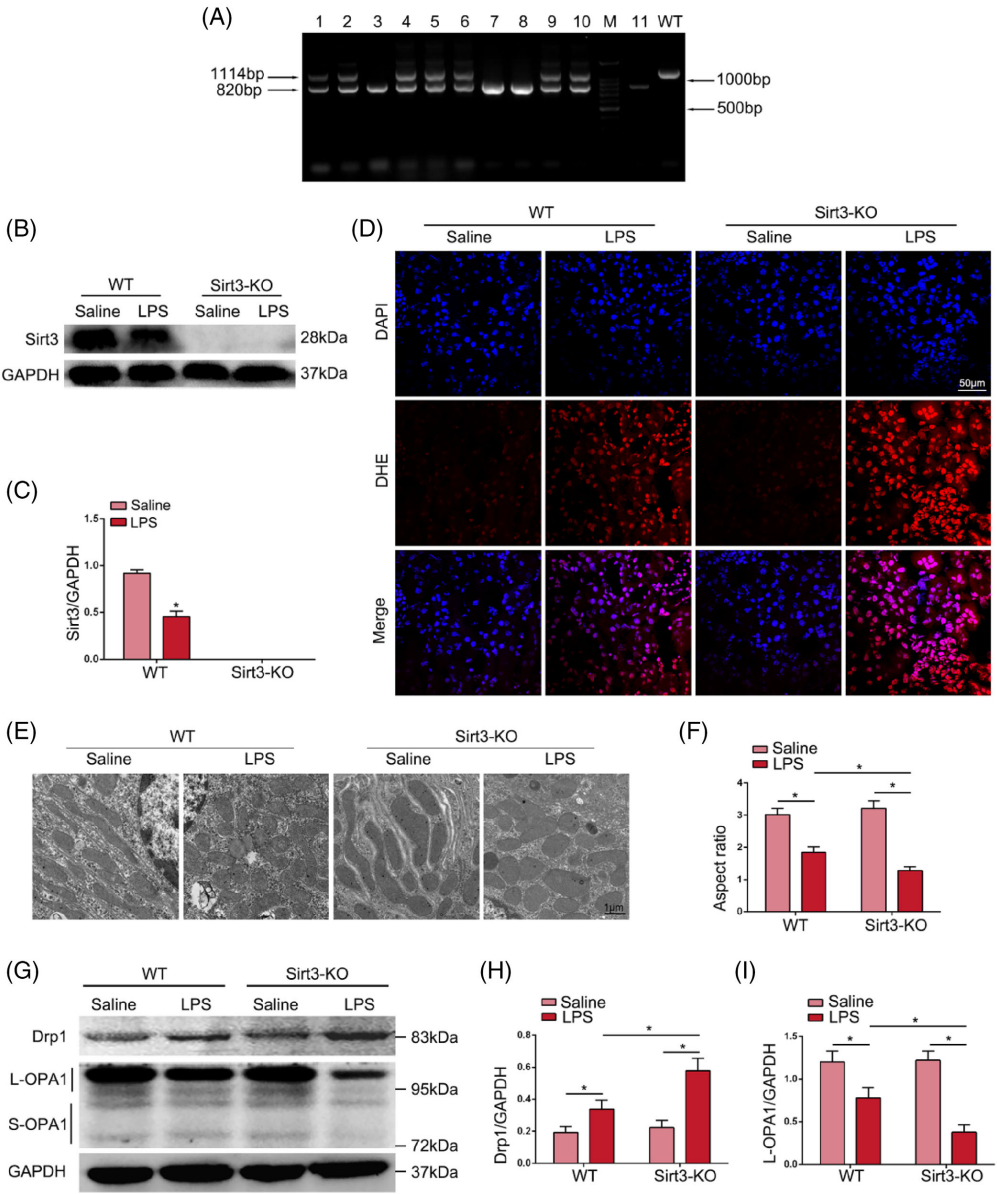
Sirt3 deficiency aggravated the abnormality of mitochondrial dynamics and functions in septic AKI mice. (A) The genotypes of transgenic mice were determined using PCR. (B, C) Representative western blots of Sirt3 expression and western blot quantification in renal tissues (*n* = 6). (D) DHE staining evaluated ROS production in TECs of mice (original magnification, ×600). DAPI (blue) was used to stain the cell nucleus. Scale bar = 50 μm. (E, F) The mitochondrial morphology of TECs in each group was observed by electron microscopy and the aspect ratio of mitochondria was performed for semiquantitative evaluation (*n* = 6) (original magnification, ×8000). Scale bar = 1 μm. (G, H) Representative western blots of Drp1 and OPA1 expression and western blot quantification in renal tissues (*n* = 6). *p < 0.05.

To further examine the role of Sirt3 in TECs in septic AKI mice, Sirt3‐KO mice were constructed. To determine the genotypes, PCR products were examined by agarose gel electrophoresis. In Sirt3‐KO mice, only the 820‐bp band was visualized (Figure 4A). Western blot analysis showed that the expression of Sirt3 was downregulated by LPS, whereas there was no expression of Sirt3 in Sirt3‐KO mouse kidney tissues (Figure 4B,C). DHE staining showed that LPS‐induced ROS production in septic AKI was further increased in the renal tissues of Sirt3‐KO mice (Figure 4D). Electron microscopy revealed that Sirt3 deficiency exacerbated the mitochondrial morphological changes induced by LPS in the renal tissues of mice, and the mitochondrial aspect ratio was further reduced (Figure 4E,F). High levels of Drp1 and low levels of L‐OPA1 in renal tissues were induced by LPS in Sirt3‐KO mice compared with the effect of saline. Moreover, knockout of the Sirt3 gene further upregulated Drp1 protein levels and downregulated L‐OPA1 in response to LPS (Figure 4G,I). These results indicated that Sirt3 deficiency exacerbated the LPS‐induced abnormalities in mitochondrial dynamics and function in renal tissues.

### 3.5 Sirt3 deficiency exacerbates TEC damage and apoptosis

**FIGURE 5:**
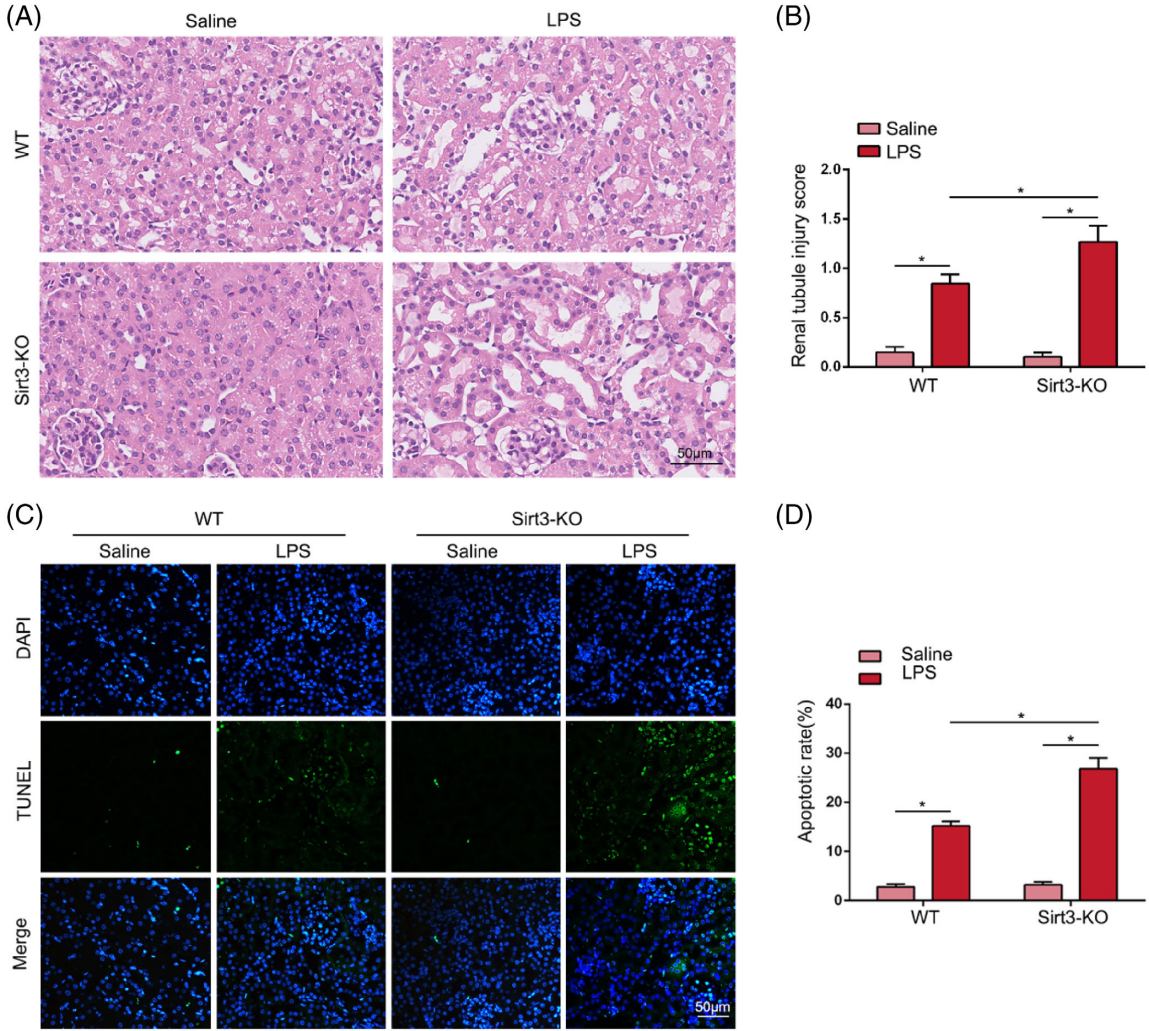
Sirt3 deficiency exacerbated LPS‐induced renal damage and TEC apoptosis of mice. (A, B) HE staining was utilized to observe the renal damage in each group, and the tubular damage score was performed (*n* = 6) (original magnification, ×400). Scale bar = 50 μm. (C, D) TUNEL staining indicated the apoptosis of TECs in each group and the percentage of apoptotic cells were performed (*n* = 6) (original magnification, ×400). Scale bar = 50 μm. *p < 0.05.

After LPS stimulation, wild‐type mice showed vacuolar degeneration, TEC detachment and partial tubular lumen dilatation, while the pathological damage to the renal tubules in the Sirt3‐KO group was further exacerbated (Figure 5A,B). TUNEL staining indicated that LPS‐induced apoptosis in TECs in septic AKI mice was further increased by Sirt3 deficiency (Figure 5C,D).

### 3.6 Overexpression of Sirt3 alleviates LPS‐induced apoptosis and mitochondrial injury in HK‐2 cells

**FIGURE 6:**
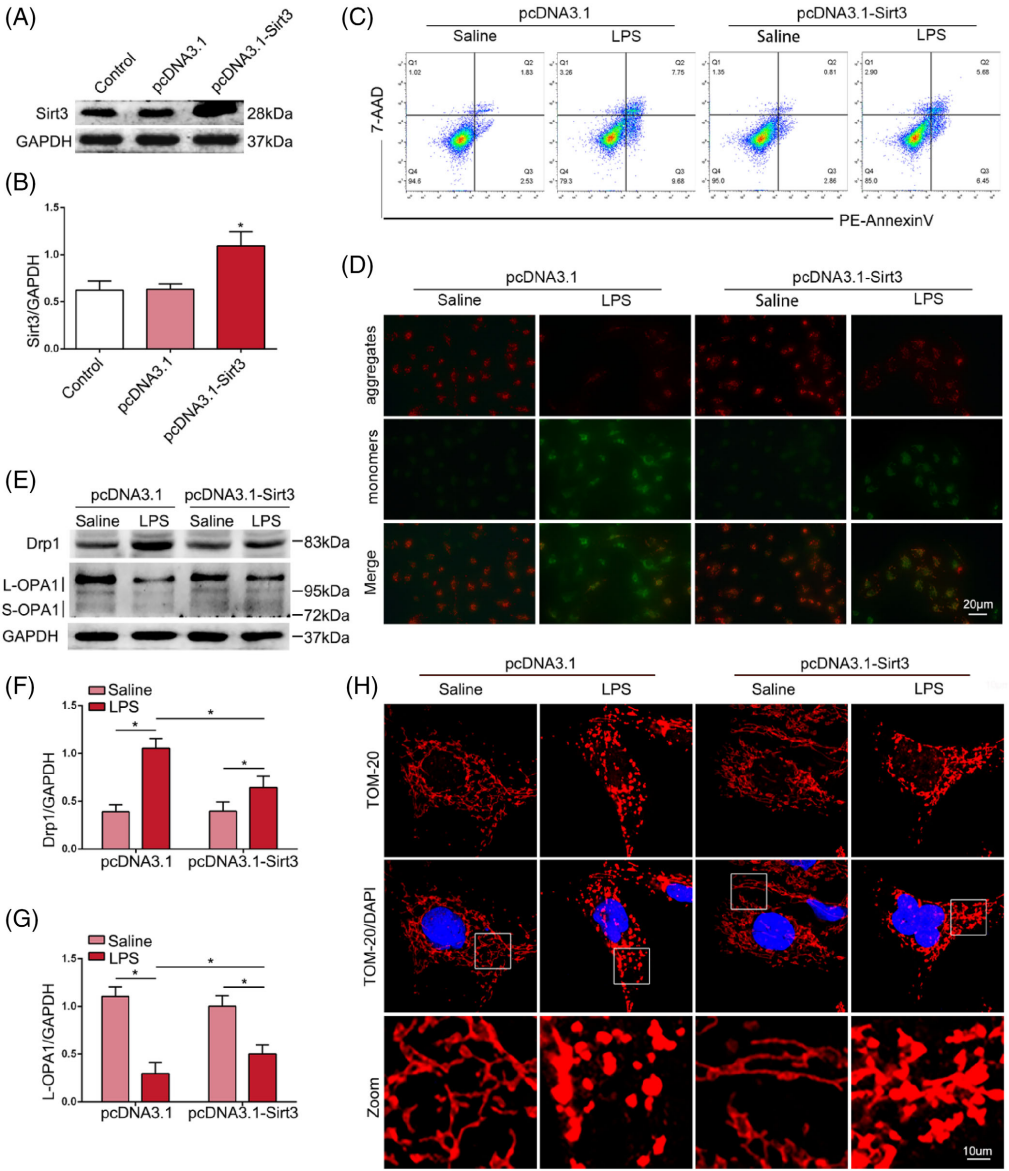
Overexpression of Sirt3 alleviated LPS‐induced apoptosis and mitochondrial injury of HK‐2 cells. (A, B) Sirt3 overexpression in HK‐2 cells with pcDNA3.1‐Sirt3 transfection was confirmed by western blot (*n* = 4). (C) Flow cytometric assessment of apoptosis in each group. (D) Representative photomicrographs of JC‐1 staining (original magnification, ×200). Red fluorescence represents JC‐1 aggregates. Green fluorescence represents JC‐1 monomers. Scale bar = 20 μm. (E–G) Representative western blots of Drp1 and OPA1 expression and western blot quantification in HK‐2 cells (*n* = 4). (H) Mitochondrial morphology in HK‐2 cells was subjected to IF‐staining using TOM‐20 antibody (original magnification, ×1000). Scale bar = 10 μm. *p < 0.05.

To further investigate the role of Sirt3 in LPS‐induced mitochondrial injury in TECs, the overexpression plasmid for Sirt3 was transfected into HK‐2 cells. Western blot analysis showed that the protein level of Sirt3 in HK‐2 cells was increased by pcDNA3.1‐Sirt3 plasmid transfection (Figure 6A,B). Flow cytometry demonstrated that LPS‐induced apoptosis in HK‐2 cells was significantly decreased by Sirt3 overexpression (Figure 6C). LPS‐challenged HK‐2 cells had reduced JC‐1 aggregates and increased JC‐1 monomers, indicating LPS‐induced unhealthy mitochondrial depolarization. The lesions were alleviated by Sirt3 overexpression (Figure 6D). Western blot analysis showed that the upregulation of Drp1 and the downregulation of L‐OPA1 induced by LPS were inhibited by Sirt3 overexpression (Figure 6E–G). The immunofluorescent staining of TOM20 showed that the continuous mitochondrial network was disrupted by LPS stimulation, while Sirt3 overexpression alleviated mitochondrial morphological changes (Figure 6H). These results indicated that Sirt3 overexpression alleviated LPS‐induced abnormal mitochondrial function and dynamics in HK‐2 cells.

### 3.7 The acetylation of YME1L1 regulated OPA1‐mediated mitochondrial fusion

**FIGURE 7:**
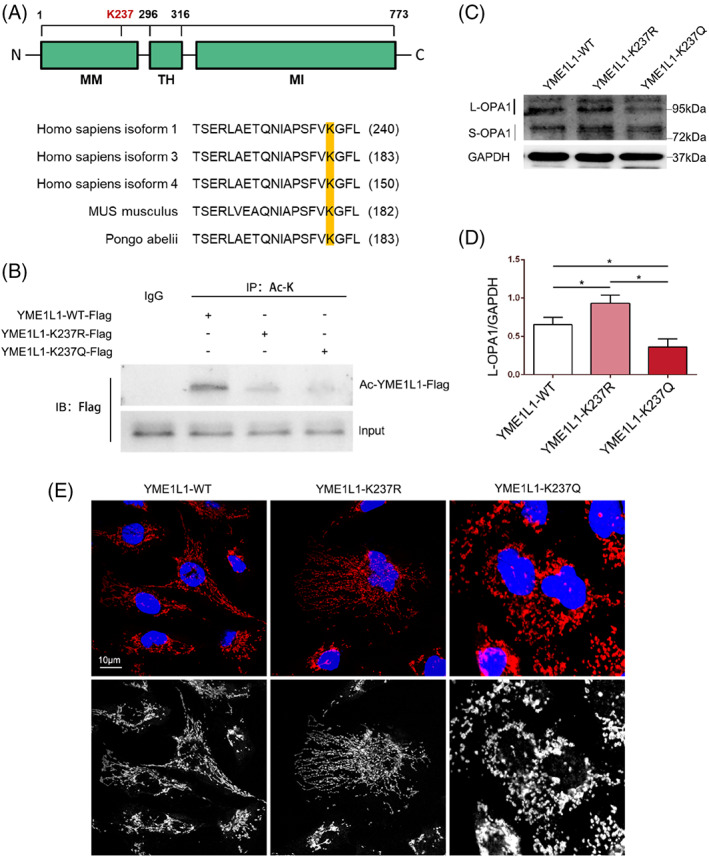
The acetylator status of YME1L1 regulated OPA1‐mediated mitochondrial fusion. (A) The amino acid sequence of YME1L1 contains lysine (K) site in various species (human, mouse and orangutan) queried in NCBI. (B) Immunoprecipitation detected the labelling on lysine in each group with control IgG as a negative control. (C, D) The protein levels of OPA1 in each group were detected using a western blot with GAPDH as a reference (*n* = 4). *p < 0.05. (E) Mitochondrial morphology in HEK‐293 cells was subjected to IF‐staining using TOM‐20 antibody (original magnification, ×1000). Scale bar = 10 μm.

YME1L1 regulates the balance between L‐OPA1 and S‐OPA1, which anchor to the mitochondrial inner membrane through their transmembrane domains. A growing number of studies have confirmed that YME1L1‐mediated processing of OPA1 is required to increase mitochondrial fusion.^26^, ^27^ Based on the important regulatory role of Sirt3 in mitochondrial dynamics and its powerful deacetylation activity, we hypothesize that Sirt3 plays a protective role in LPS‐induced TECs injury by regulating OPA1‐mediated mitochondrial fusion by deacetylating YME1L1. The amino acid sequence of YME1L1 was queried in NCBI. The results showed that the amino acid sequence of YME1L1 contains a lysine (K) site in various species (human, mouse and orangutan), which is located at position 237 in human isoform 1, indicating a conserved site (Figure 7A). To further confirm that the acetylation status of YME1L1 affects OPA1‐mediated mitochondrial fusion, we altered the acetylation status of YME1L1 by transfecting gene mutant plasmids. The YME1L1‐K237R plasmid mutated lysine 237 (K) to arginine (R) to mimic the deacetylated state, and the YME1L1‐K237Q plasmid mutated lysine 237 (K) to glutamine (Q) to mimic the acetyl state. No labelling of lysine was detected in HEK‐239 cells after transfection with mutant YME1L1 K237R or mutant YME1L1 K237Q plasmids by IP, which revealed that the construction of the mutant plasmids was successful (Figure 7B). Western blot results showed that the expression of L‐OPA1 was increased in HEK‐293 cells transfected with the YME1L1‐K237R plasmid compared with cells transfected with the YME1L1‐WT plasmid, and it was decreased in HEK‐293 cells transfected with the YME1L1‐K237Q plasmid (Figure 7C,D). IF staining of the mitochondrial marker TOM‐20 revealed that the mitochondria of HEK‐293 cells transfected with the YME1L1‐K237R plasmid showed a closer network structure than those transfected with the YME1L1‐WT plasmid, while the mitochondria of HEK‐293 cells transfected with the YME1L1‐K237Q plasmid showed obvious mitochondrial fission (Figure 7E). These results confirmed that the acetylation of YME1L1 inhibited mitochondrial fusion and promoted mitochondrial fission, while deacetylation promoted mitochondrial fusion and inhibited mitochondrial fission.

### 3.8 Sirt3 regulates OPA1‐mediated mitochondrial fusion by deacetylating YME1L1 in TECs

**FIGURE 8:**
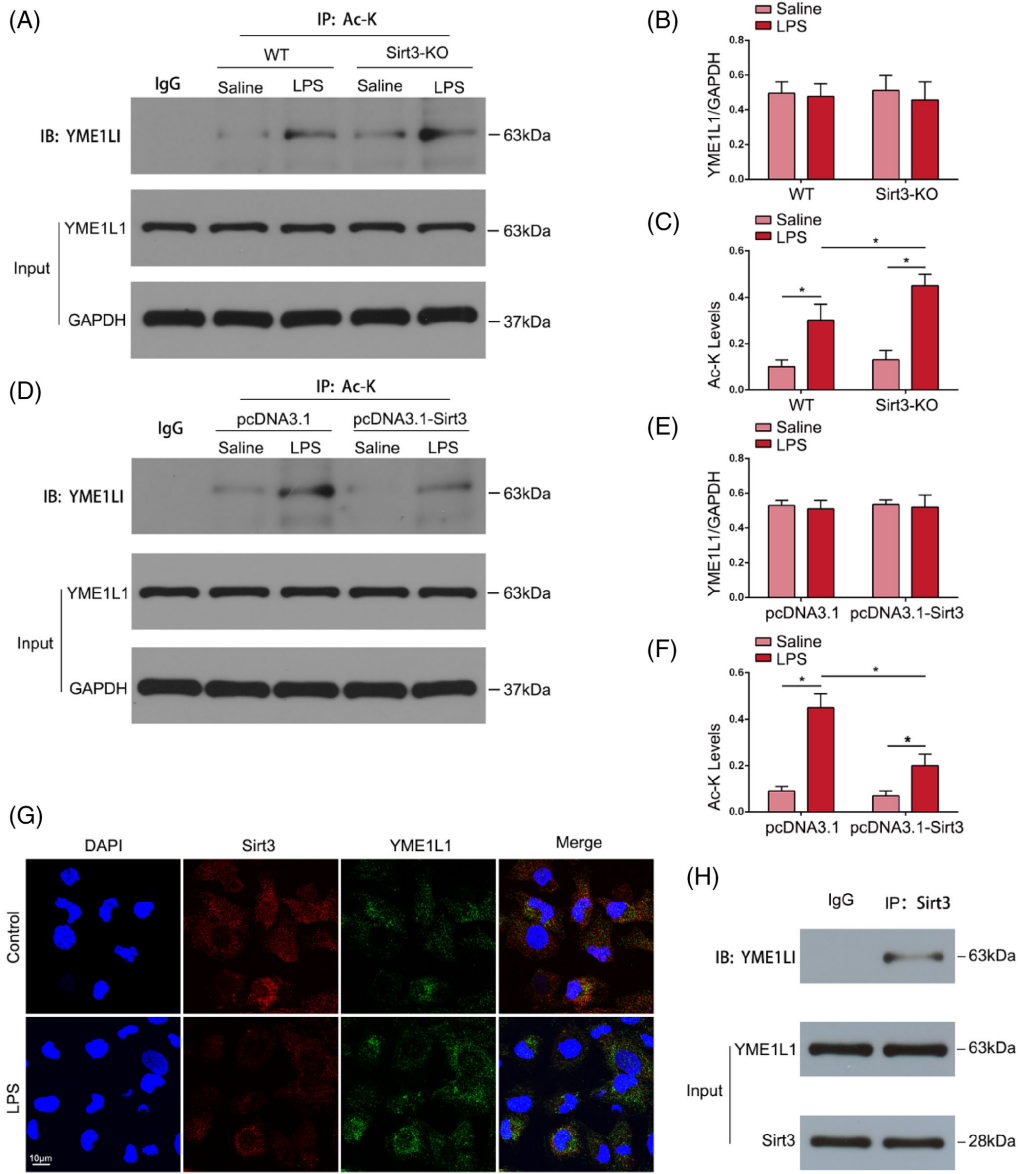
Sirt3 regulated the deacetylation of YME1L1. (A–C) Representative immunoprecipitation (IP) results of the acetylation level of YME1L1 in renal tissues and semiquantification of these results (*n* = 6). *p<0.05. (D–F) Representative IP results of the acetylation level of YME1L1 in HK‐2 cells and semiquantification of these results (*n* = 4). *p < 0.05. (G) IF staining revealed the fluorescence co‐localization between Sirt3 and YME1L1 (original magnification, ×600). Scale bar = 10 μm. (H) The interaction between Sirt3 and YME1L1 was detected by IP.

To examine whether Sirt3 regulates OPA1‐mediated mitochondrial fusion by deacetylating YME1L1 in TECs treated with LPS, we measured the protein levels of YME1L1 and its interaction with Sirt3 in TECs. Western blot and IP results showed that the total expression of YME1L1 in the LPS‐induced septic AKI mouse model did not change significantly compared with that in the control group, but the acetylation level was increased, while the acetylation level of YME1L1 was further increased by Sirt3 deletion (Figure 8A–C). Moreover, the total expression of YME1L1 in HK‐2 cells did not change significantly, but the acetylation level increased in response to LPS, and the acetylation level of YME1L1 was decreased by Sirt3 overexpression (Figure 8D,F). Both Sirt3 and YME1L1 are expressed in the mitochondria of TECs. IF staining indicated that there was colocalization of Sirt3 and YME1L1 in normal cultured HK‐2 cells, but this colocalization was weakened by LPS (Figure 8G). The IP results showed that Sirt3 and YME1L1 interacted in HK‐2 cells (Figure 8H). These results suggested that Sirt3 deacetylated YME1L1 and further confirmed that Sirt3 had a protective role in LPS‐induced TECs injury by regulating OPA1‐mediated mitochondrial fusion by deacetylating YME1L1.

## 4 DISCUSSION

Sepsis is the most frequent cause of AKI in critically ill patients, and septic AKI is associated with high short‐term mortality.^3^, ^28^ Persistent and excessive renal tissue damage and inflammation result in the failure of restoration. A considerable proportion of AKI patients develop chronic renal failure, which eventually progresses to end‐stage renal disease.^29^ Although substantial investigations on AKI have been performed, the mechanisms by which sepsis induces AKI remain incompletely understood, and numerous clinical therapeutic approaches have been unsuccessful.

In recent years, dysfunctional energy metabolism in TECs has been considered a pivotal cause of LPS‐induced septic AKI.^7^, ^30^ The core of renal TEC energy metabolism disorder is mitochondrial damage because damaged mitochondria cannot normally capture electrons to complete OXPHOS, resulting in excessive production of ROS and an insufficient supply of ATP.^31^ Studies have shown that during renal TEC apoptosis induced by sepsis, mitochondrial numbers are reduced, cristae fragment and membrane integrity is impaired, and reducing mitochondrial damage can reverse TECs apoptosis.^32^ These findings indicate that mitochondrial damage is closely related to apoptosis in septic AKI.

The mitochondrial network maintains a dynamic balance between fusion and fission, which is also known as mitochondrial dynamics.^11^, ^33^ The morphological changes in mitochondria are closely related to their function. Under excessive noxious stimulation, mitochondrial fission increases, the mitochondrial network is damaged, more small mitochondria are produced, and these mitochondria are more susceptible to oxidative stress damage, resulting in insufficient ATP production, while increased mitochondrial fusion can generate tighter mitochondrial networks to fight oxidative damage and produce more ATP.^34^ In the present study, we observed decreased fusion, increased division, and increased mitochondrial ROS production in TECs after LPS stimulation, suggesting that LPS induced abnormal mitochondrial morphology and function in TECs. Fusion and fission are regulated by guanosine triphosphatases (GTPases), of which Drp1 and OPA1 are the most important molecules. Drp1 is distributed in the cytoplasm and is recruited to the outer membrane of mitochondria to drive mitochondrial fission in response to noxious stimuli,^35^ and OPA1 is anchored to the mitochondrial inner membrane mediates mitochondrial fusion and is involved in maintaining mitochondrial crest morphology.^36^ Studies have shown that OPA1 can mediate mitochondrial fusion, coordinate mitochondrial crest morphology and resist apoptosis.^37^, ^38^ The fusion activity of OPA1 relies on the presence of both L‐OPA1 and S‐OPA1 forms, especially L‐OPA1. Although S‐OPA1 is also essential for fusion, its excessive accumulation may promote mitochondrial fission.^14^ In this study, we found that disruption of the complete mitochondrial network was accompanied by reduced L‐OPA1.

Sirt3 is the main mitochondrial deacetylase and is involved in most mitochondrial‐related biological processes by regulating the deacetylation of related key proteins.^39^ Studies have shown that the level of mitochondrial protein acetylation is increased significantly by Sirt3 deletion.^40^ Accumulating evidence has shown that Sirt3 can relieve injury due to metabolic stress, tumours, the development of cardiac hypertrophy and age‐related hearing loss by reducing mitochondrial damage and ROS production.^41^ Another study showed that Sirt3 expression was downregulated in cisplatin‐induced AKI, and restoring Sirt3 expression could protect TECs by reducing abnormal mitochondrial dynamics.^42^ In a septic AKI model induced by caecal ligation and puncture, a study showed that restoring Sirt3 expression could reduce mitochondrial damage by inhibiting inflammasomes, downregulating interleukin levels and reducing oxidative stress levels.^24^ Consistent with previous studies, we found increased short and small mitochondria, increased Drp1 protein expression and decreased L‐OPA1 protein expression in TECs after LPS stimulation, all of which were further exacerbated in Sirt3‐KO mice, suggesting that Sirt3 deletion exacerbated LPS‐induced abnormal mitochondrial dynamics in TECs. In contrast, these phenomena were significantly alleviated in Sirt3‐overexpressing HK‐2 cells, suggesting that Sirt3 overexpression alleviated LPS‐induced abnormal mitochondrial dynamics in TECs. However, the specific mechanism by which Sirt3 regulates mitochondrial dynamics and function needs further study.

OPA1‐mediated mitochondrial fusion is regulated by two mitochondrial proteases, the i‐AAA protease YME1L1 and the m‐AAA protease OMA‐1, which are both involved in the hydrolysis of OPA1 and are considered to be central to mitochondrial fusion.^43^ OMA1 and YME1L cleave L‐OPA1 (a and b) at S1 and S2, respectively, to yield S‐OPA1 forms (c, d, and e).^14^ Constitutive processing of L‐OPA1 by YME1L1 at S2 and by OMA1 at S1 generates a mix of L‐OPA1 and SOPA1 in healthy cells.^44^ However, in pathological states, L‐OPA1 are over‐cleaved to S‐OPA1, which results in reduced mitochondrial fusion and increased mitochondrial division. Numerous investigations have confirmed that YME1L1‐mediated OPA1 processing is necessary to improve the mitochondrial fusion rate.^45^ For example, Anand et al^27^ showed that S‐OPA1 production was significantly increased in mouse embryonic fibroblasts with YME1L1 deficiency, suggesting that the lack of YME1L1 may accelerate the hydrolysis of OPA1 by OMA1, while the loss of OMA1 prevented the formation of S‐OPA1 in cells and restored tubular mitochondrial morphology. Although YME1L1‐mediated cleavage of L‐OPA1 is essential for mitochondrial fusion, relatively little is known about the effect of increased YME1L1 activity on mitochondrial morphological changes. Yang et al. indicated that Sirt3 could reduce mitochondrial damage by regulating the deacetylation of mitochondrial matrix proteins under stress conditions.^46^ We wondered whether Sirt3 plays a protective role in LPS‐induced renal TEC injury by regulating OPA1‐mediated mitochondrial fusion by deacetylating YME1L1 or OMA1. According to the literature, Cheng et al. recently examined T‐cell memory and showed that sugar restriction promoted T‐cell mitochondrial fusion and ATP generation by increasing the deacetylase activity of Sirt3. Further mechanistic studies showed that Sirt3 deacetylates YME1L1 leading to suppression of OPA1 processing, which promotes mitochondrial fusion, and they found that Sirt3 can interact with YME1L1 but not OMA1.^25^, ^46^ To further examine the hypothesis that the regulatory effect of Sirt3 on septic AKI depends on mediating the deacetylation of YME1L1, the expression of YME1L1 and acetylated YME1L1 was assessed. We found that the acetylation level of YME1L1 in TECs was increased by LPS stimulation, Sirt3 gene knockout further increased the acetylation level of YME1L1, and Sirt3 overexpression decreased the acetylation level of YME1L1, suggesting that Sirt3 deacetylated YME1L1. To further confirm the effect of YME1L1 acetylation status on OPA1‐mediated mitochondrial fusion, we established mutant YME1L1 acetylation or deacetylation mimetics, our results showed that YME1L1 acetylation increased L‐OPA1 processing, thereby inhibiting mitochondrial fusion. Finally, we also confirmed the interaction between Sirt3 and YME1L1 by IP. These data reveal that Sirt3 deacetylates YME1L1 to promote OPA1‐mediated mitochondrial fusion by suppressing the processing of L‐OPA1.

In summary, the Sirt3‐YME1L1‐OPA1 signalling pathway may be an important factor in mitochondrial damage in TECs in LPS‐induced septic AKI. Our investigation provides a theoretical basis for the prevention and treatment of septic AKI.

## AUTHOR CONTRIBUTIONS

Yonghong Jian, Yifei Yang and Dingping Yang conceived and designed the experiments. Yonghong Jian and Yifei Yang performed the main experiments, analysed the data and drafted the manuscript. Lingli Cheng, Xueyan Yang, Hongyan Liu, Wei Li and Yuhan Wan participated in some experiments. Dingping Yang revised the manuscript. All authors have read and approved the final manuscript.

## CONFLICT OF INTEREST

No potential conflict of interest was reported by the authors.

## Data Availability

Data supporting the findings of this study are available from the corresponding author upon reasonable request.
